# Inactivation of the NLRP3 inflammasome mediates exosome-based prevention of atrial fibrillation

**DOI:** 10.7150/thno.89520

**Published:** 2024-01-01

**Authors:** Sandrine Parent, Ramana Vaka, Jennifer St Amant, Saad Kahn, Sophie Van Remortel, Christina Bi, David Courtman, Duncan John Stewart, Darryl Raymond Davis

**Affiliations:** 1 University of Ottawa Heart Institute, Division of Cardiology, Department of Medicine, University of Ottawa, Ottawa, CANADA K1Y4W7.; 2 Department of Cellular and Molecular Medicine, Faculty of Medicine, University of Ottawa, Ottawa, Canada, K1H8M5.; 3 Ottawa Hospital Research Institute, Regenerative Medicine Program, Ottawa, Canada, K1H8L6.

**Keywords:** exosomes, extracellular vesicles, atrial fibrillation, postoperative, NLRP3

## Abstract

**Rationale:** Extracellular vesicles (EVs) from human explant-derived cells injected directly into the atria wall muscle at the time of open chest surgery reduce atrial fibrosis, atrial inflammation, and atrial fibrillation (AF) in a rat model of sterile pericarditis. Albeit a promising solution to prevent postoperative AF, the mechanism(s) underlying this effect are unknown and it is not clear if this benefit is dependent on EV dose.

**Methods:** To determine the dose-efficacy relationship of EVs from human explant-derived cells in a rat model of sterile pericarditis. Increasing doses of EVs (10^6^, 10^7^, 10^8^ or 10^9^) or vehicle control were injected into the atria of middle-age male Sprague-Dawley rats at the time of talc application. A sham control group was included to demonstrate background inducibility. Three days after surgery, all rats underwent invasive electrophysiological testing prior to sacrifice.

**Results:** Pericarditis increased the likelihood of inducing AF (p<0.05 vs. sham). All doses decreased the probability of inducing AF with maximal effects seen after treatment with the highest dose (10^9^, p<0.05 vs. vehicle). Pericarditis increased atrial fibrosis while EV treatment limited the effect of pericarditis on atrial fibrosis with maximal effects seen after treatment with 10^8^ or 10^9^ EVs. Increasing EV dose was associated with progressive decreases in pro-inflammatory cytokine content, inflammatory cell infiltration, and oxidative stress. EVs decreased NLRP3 (NACHT, LRR, and PYD domains-containing protein-3) inflammasome activation though a direct effect on resident atrial fibroblasts and macrophages. This suppressive effect was exclusive to EVs produced by heart-derived cells as application of EVs from bone marrow or umbilical cords did not alter NLRP3 activity.

**Conclusions:** Intramyocardial injection of incremental doses of EVs at the time of open chest surgery led to progressive reductions in atrial fibrosis and inflammatory markers. These effects combined to render atria resistant to the pro-arrhythmic effects of pericarditis which is mechanistically related to suppression of the NLRP3 inflammasome.

## Introduction

Despite more than half a century of experience, 30-50% of patients still experience atrial fibrillation (AF) 2-3 days after cardiac surgery 1-11. AF impacts postoperative healing as rapid uncontrolled heart rates place metabolic demands on the post-surgical heart 10, 12-14 while the immobile atria increase the risk of clot formation and stroke 3, 14-19. As such, postoperative AF increases hospital costs 6, lengths of stay 1, 3, 6, 15, 17, 20-22, and patient morbidity 10, 12-14. Aside from continuing beta-blockers to suppress the ectopic beats that trigger AF, there are no medications or procedures that conclusively reduces the occurrence of AF after cardiac surgery 23.

In response to this challenge, we explored the ability of pro-healing extracellular vesicles (EVs) to reduce AF in a rat model of sterile pericarditis 24. EVs are lipid enclosed microparticles that bud off from the membrane of cells to communicate with neighbouring tissue via fusion and uptake of proteins or non-coding RNAs. EVs can be isolated from media conditioned by cardiac explant-derived cells (EDCs) for direct application as an acellular therapy. Cardiac EDCs are CD45- CD105+ cells cultured directly from atrial biopsies for therapeutic application 25-27. In a manner consistent with effects seen in post infarct recovery models, we found that injection of a single dose of EVs into atrial tissue at the time of open chest surgery reduced inflammation and rendered resident fibroblasts resistant to pro-fibrotic stimuli. Most importantly, this pre-conditioning effect reduced the incidence of AF back to baseline (absolute risk reduction = 48%, relative risk reduction = 66% and number needed to treat = 2).

Mechanistically, intra-myocardial injected EVs appear to work primarily on resident atrial fibroblasts and macrophages. Treated fibroblasts are reprogrammed to slow proliferation and resist stimuli inductive from inflammatory cytokines. Macrophages are similarly modified to adopt a pro-healing phenotype which discourages infiltration of inflammatory cells. These actions reduce AF vulnerability whereupon any ectopic beats cannot trigger re-entrant arrhythmias. Despite this insight, it is not known if these effects occur as simultaneous independent events or in a reproducible, stereotypical sequence. It is also not clear if a minimally effective “dose” exists whereby exposure to EVs reduce AF inducibility or if this benefit is incrementally dependent on the “dose” of EVs applied. Finally, it is uncertain what pathway drives the observed salutary effects. The latter being particularly important as understanding what pathways underlie this effect will operationalize the identification and development of more effective therapies.

As such, we designed a study to determine the dose-efficacy relationship of EVs produced by human EDCs in a rat model of sterile pericarditis. Study outcomes focused on measures associated with the development of postoperative AF. Given that EVs represent a biological product amenable to targeted engineering of the producer cell line, understanding these relationships are important as future efforts could be tailored to enhance therapeutic potency.

## Materials and Methods

### Cell culture and EV isolation

All cell products were manufactured to clinical grade release standards in Biospherix units at The Ottawa Hospital Cell Manufacturing Facility 25. Human cardiac EDCs were isolated from atrial appendages collected during cardiac surgery after informed consent using a protocol approved by the University of Ottawa Heart Institute Research Ethics Board. EDCs were cultured using serum-free, xenogen-free methods in NutriStem XF media (Sartorius) under constant 5% oxygen conditions 25-27. Bone marrow mesenchymal stromal cells (BM-MSCs) were isolated from bone marrow aspirates from healthy volunteers collected under the Ottawa Hospital Research Ethics Board approved Cellular Immunotherapy for Septic Shock (CISS) trial 28. BM-MSCs were cultured in NutriStem XF media (Sartorius) under 21% oxygen conditions 28. Umbilical cord mesenchymal stromal cells (UC-MSCs) were isolated from umbilical cords collected during scheduled C-sections performed at The Ottawa Hospital under protocols approved by Ottawa Hospital Research Ethics Board 29, 30. UC-MSCs were cultured in high glucose Dulbecco's Modified Eagle Medium (ThermoFisher Scientific) with 10% clinical grade platelet lysate (Mill Creek Life Sciences) at 5% oxygen conditions. Condition media was generated over 48 hours of culture at 1% oxygen conditions using NutriStem XF basal media (BM-MSCs and EDCs) or Dulbecco's Modified Eagle Medium with high glucose and 1% platelet lysate (UC-MSCs). EVs were isolated using ultracentrifugation (10,000g X 30 minutes and 100,000g X 3 hours) 31, 32. The details of EDC EV characterization have been submitted to the EV-TRACK knowledgebase (EV-TRACK ID: EV210347) 33, 34.

THP-1 cells (TIB-202, American Type Culture Collection) were cultured in Roswell Park Memorial Institute 1640 (RPMI1640, Signa Aldrich) supplemented with 10% fetal bovine serum (Thermo Fischer Scientific) prior to differentiating into macrophages using 100 nM phorbol-myristate acetate (PMA, Thermo Fischer Scientific) for 3 days.

Primary atrial fibroblasts were isolated from 6-month-old female Sprague Dawley rats using enzymatic digestion (Collagenase Type II, Worthington Biochemical). Cells were cultured in Dulbecco's Modified Eagle high glucose medium, supplemented with 10% fetal bovine serum (Thermo Fischer Scientific), 1% l-glutamine (Thermo Fischer Scientific), and 1% penicillin-streptomycin (Thermo Fischer Scientific).

### In vivo efficacy study

Female Sprague Dawley rats (6 months old, Charles River) underwent induction of sterile pericarditis or sham operation under a protocol approved by the University of Ottawa Animal Care Committee. We elected to focus on female recipients as previous work has shown recipient sex did not alter the ability of EVs to prevent inducible AF.

Rats were housed in an accredited animal facility. Prior to surgery, all rats were induced with buprenorphine (0.03 mg/kg subcutaneous) prior to anesthesia (3% isoflurane), intubated, and ventilated. Using a sealed envelope approach, animals were randomized to increasing doses of EVs (10^6^, 10^7^, 10^8^ or 10^9^, n=10-20/group) or vehicle control (n=10) injected into the atria of rats at the time of talc application. A sham control group was included to demonstrate background inducibility (n=14). Intramyocardial injections were performed using a Hamilton microsyringe (27-gauge needle) into the left atrial wall at 6 separate injection points 35. After surgery, animals recovered in a 30-degree Celsius incubator with supplemental oxygen and moistened food. All animals received buprenorphine (0.03 mg/kg subcutaneous) 6 and 12 hours after the operation. Lab staff were blinded to the treatment received and analysis was conducted by individuals blinded to group allocation. Group allocations were kept in a separate password protected list for unblinding after analysis of the primary study outcome was completed.

All rats underwent invasive electrophysiological testing 3 days after surgery 35. Anesthesia was induced using intraperitoneal injection of sodium pentobarbital (40 mg/kg) prior to insertion of a 1.6F octopolar catheter (Millar) into the right atrium via the jugular vein for stimulation and recording. Electrophysiologic testing was performed as previously described. Briefly, atrioventricular nodal refractory period was defined as the longest S1-S2 interval that failed to conduct to the ventricle using twice-threshold, 2-ms, square-wave pulses after a 10-stimulus drive train (S1, 100-ms cycle length) followed by an S2 decremented in 2-ms intervals. A minimum of ten cycles of atrial burst pacing that lasted 5-30 seconds was then performed at cycle lengths that ranged between 20 and 80 milliseconds to test AF inducibility. AF was defined as rapid and fragmented atrial electrograms, the absence of discernible P waves on the surface electrocardiogram and an irregular ventricular rhythm that lasted for at least 500 ms 36. AF duration was defined as the longest single episode recorded. This data is displayed as the average of the longest episodes of AF for each group of animals. To probe for an effect of EV treatment on the duration of AF, animals that did not experience AF were not included in this average. At the end of the study, rats were sacrificed by exsanguination after displaying absence of withdrawal reflex to toe pinch.

### Histological analysis, quantification of fibrosis and markers of fibrotic turnover

Atria were collected, fixed, and sectioned for histological analysis of alpha smooth muscle actin (αSMA; A5228, Sigma), inflammatory infiltrates (hematoxylin and eosin (H&E), Sigma), collagen (ab210579, Abcam; LS-F5563-1, LifeSpan BioSciences; picrosirius red, Thermo Fisher Scientific), wheat germ agglutinin (Thermo Fisher Scientific), connexin 40 (ab1726, Millipore Sigma), and connexin 43 (C6219, Sigma). Images were quantified using ImageJ 37, 38. Left atria collagen content was confirmed using an assay for hydroxyproline (ab222941, Abcam), an indicator of collagen content/turnover 39, 40.

Homogenized atrial tissue was analyzed for markers associated with RTFI00051 using enzyme-linked immunosorbent assays (ELISAs). These assays included matrix metalloproteinase 2 (MMP2; RTFI00042, AssayGenie), MMP9 (RTFI00080, AssayGenie), tissue inhibitor of metalloproteinases 2 (TIMP2; RTFI00051, AssayGenie), TIMP3 (RTFI01169, AssayGenie), and αSMA (MBS2703516, MyBioSource).

### Atrial quantification for markers of inflammation and myeloperoxidase activity

Homogenized atrial tissue obtained after the invasive electrophysiological study was analyzed for markers of atrial inflammation using ELISAs and a custom multiplex Luminex-based assay (LXSARM, R&D Systems). Interleukin 6 (IL-6, ERA32RB, Life Sciences), IL-17 (MBS2022678, MyBioSource), platelet-derived growth factor AB (PDGF-AB, ab213906, Abcam), monocyte chemoattractant protein-1 (MCP-1, ab100778, Abcam) and transforming growth factor beta 1 (TGFβ1; ab119558, Abcam) were analyzed using commercially available ELISAs. Four analytes (IL-1β, IL-10, IL-18, tumor necrosis factor alpha (TNFα)) were measured using a MAGPIX system (LXSARM-06) with post-hoc mean fluorescence quantification using xPONENT software. Atrial myeloperoxidase activity was evaluated using a colorimetric assay according to the manufacturer's directions (ab105136, Abcam).

### NLRP3 inflammasome activity

NLRP3 inflammasome activity and downstream mediators of NLRP3 activation was evaluated in rat atrial fibroblasts, THP-1 macrophages, and atrial tissue lysates. Rat atrial fibroblasts and THP-1 macrophages were treated with BM-MSC, EDC or UC-MSC EVs before exposure to 500 ng/mL lipopolysaccharide (LPS, L4391-1MG, Sigma) and 20 µM nigericin (N7143-5MG, Sigma) to activate the NLRP3 inflammasome. A bioluminescent assay was used to evaluate secreted caspase-1 activity (G9951, Promega) while IL-1β and IL-18 in culture supernatants and tissue lysates were quantified using ELISA (IL-1β; DY201-05 & IL-18; DY318-05, R&D) and Luminex discovery assay (LXSARM-06, R&D) respectively. The specificity of caspase-1 activation was validated by including the caspase-1 inhibitor (YVAD-CHO, Promega). NLRP3 activity was evaluated in atrial tissue lysates using a commercial ELISA (ab277086, Abcam).

### Identification of NLRP3 inflammasome modulating EV miRNAs and proteins

In prior work, we isolated EVs from EDC conditioned media for profilin of the EV miRNA and protein cargo using Nanostring and Mass spectrometry, respectively 29. In this paper, we applied bioinformatic tools Tam 2.0, miRWalk, Uniprot & a Go tool to identify enriched miRNAs and proteins targeting NLRP3.

### Statistical analysis

All statistical tests used and graphical depictions of data are defined within the figure legends for the respective data panels (GraphPad Prism 9.5). All data is presented as mean ± standard error of the mean. To determine if differences existed within groups, data was analyzed by an ordinary one-way ANOVA; if such differences existed, Šídák's multiple comparisons test was used to determine the group(s) with the difference(s). In all cases, variances normality was confirmed prior to further post-hoc testing using Bartlett's test. Non-parametric outcomes were analyzed using the Kruskal-Wallis test for multiple comparisons, as appropriate. Differences in categorical measures were analyzed using a Chi Square test. A final value of P≤0.05 was considered significant for all analyses.

## Results

### Effect of increasing EV doses on atrial fibrillation inducibility and electrical remodeling

**Figure 1:**
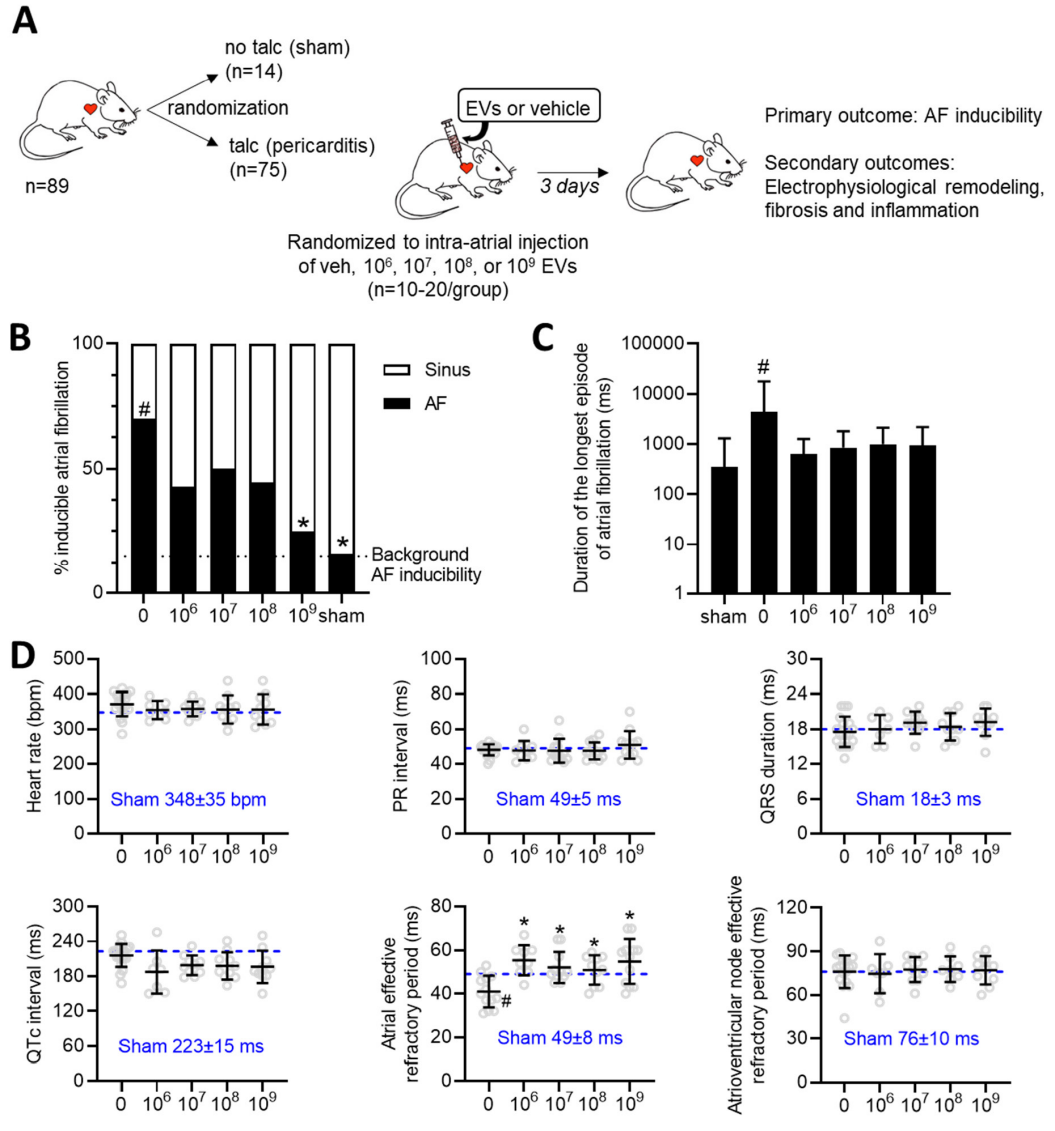
** Dose dependent effects of intramyocardial injection of human extracellular vesicles (EVs) on cardiac electrophysiology and atrial fibrillation inducibility in a model of rat sterile pericarditis.** (A) Increasing doses of EVs or vehicle control were injected into the atria of middle-age female Sprague-Dawley rats at the time of talc application. A sham control group was included to demonstrate background inducibility. Three days after surgery, all rats underwent invasive electrophysiological testing prior to sacrifice for histological and molecular characterization. (B) Effect of EV treatment on the probability of inducing AF after burst pacing. A two-sided Chi square test was performed with no penalty for multiple comparisons. *P<0.05 vs. talc + vehicle. #P<0.05 vs. sham. (C) Effect of pericarditis and EV treatment on the duration of the longest AF episode recorded. Data is displayed using a logarithmic abscissa and was analyzed using Kruskal-Wallis testing for multiple comparisons. #P<0.05 vs. sham. (D) Effect of pericarditis and EV treatment on electrocardiographic and electrophysiological function. One-way ANOVA with individual-mean comparisons by Šídák's multiple comparisons test. *P<0.05 vs. talc + vehicle. #P<0.05 vs. sham. AF, Atrial fibrillation; EPS, electrophysiological study; EV, extracellular vesicles; veh, vehicle.

The influence of increasing doses of EVs was evaluated using a validated rat model of sterile pericarditis (Figure 1A). As shown in Figure 1B, animals randomized to pericarditis and vehicle treatment demonstrated a 4-fold increase in inducible AF (70 vs. 16%, p<0.05 vs. sham). All EV doses decreased the probability of inducing AF with maximal effects seen after treatment with the highest dose (10^9^, p<0.05 vs. vehicle). Pericarditis increased the duration of the longest recorded AF episode by 5±15 seconds (p=0.028 using the Kruskal-Wallis test; Figure 1C). Although there was a trend for EV treatment to decrease the duration of the longest AF episode, this trend was not significant likely due to small number of animals used and the unequal variance nature of this outcome (p<0.0001 using Bartlett's test).

Consistent with the literature 41-44, sterile pericarditis had no effect on any of the standard electrocardiographic intervals (i.e., RR, PR, QRS or QTc interval) while reducing the effective refractory period of the atria (Figure 1D). EV administration at the time of surgery did not alter electrocardiographic intervals while attenuating the adverse effect of pericarditis on atrial refractoriness. In support of the latter, we performed electrophysiological testing on a separate cohort of rats that were simply injected with 10^9^ EVs without talc application. As illustrated in Table S1, the application of EVs had no impact on cardiac electrophysiology or atrial fibrillation inducibility; thus, refuting the notion that EVs possess an inherent antiarrhythmic effect.

**Figure 2:**
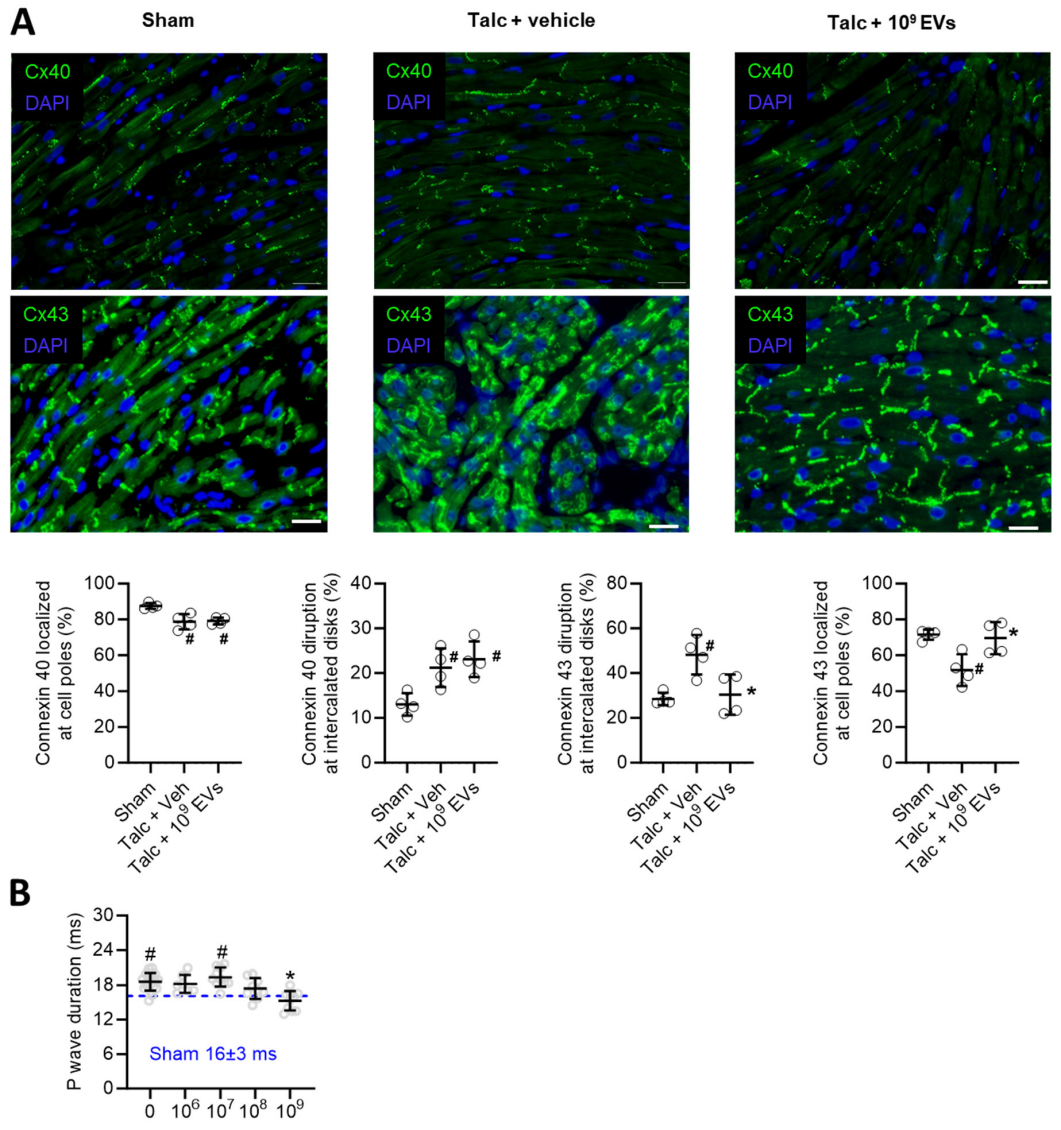
** Effects of intramyocardial injection of human extracellular vesicles (EVs) on cardiac connexin distribution and atrial activation using a model of rat sterile pericarditis.** (A) Representative images from sham, talc + vehicle and talc + EV treated animals showing connexin 40 and 43 localization at cell poles and intercalated disks (n= 4 random fields per group). Scale bar 20 μm. (B) Effect of pericarditis and EV treatment on p wave duration (n=10-19 animals per group). One-way ANOVA with individual-mean comparisons by Šídák's multiple comparisons test. *P<0.05 vs. talc + vehicle and ^#^P vs. sham.

Pericarditis causes inflammation which results in conduction abnormalities that facilitate reentry 45, 46. As shown in Figure 2A, pericarditis altered expression of connexin 40 and 43 in atrial tissue (Figure 2A). EV treatment prevented pericarditis-induced remodeling in connexin 43, but not connexin 40, which may have provided knock on benefits towards atrial conduction, but this was not tested directly in this study. Interestingly, pericarditis also increased P wave duration (Figure 2B) which reflects impaired atrial activation and was likely due to a combination of decreased connexin expression and increased atrial fibrosis (outlined below). EV treatment prevented pericarditis from altering P wave duration with maximal effects seen at the highest dose (P<0.001 vs. talc alone).

Taken together, this data suggests that, like a drug, the antiarrhythmic benefits seen with EV treatment are incrementally dependent on the “dose” applied and do not rely upon attaining a critical threshold to have salutary effects on AF induction.

### Effect of increasing EV doses on atrial markers of inflammation and oxidative stress

**Figure 3:**
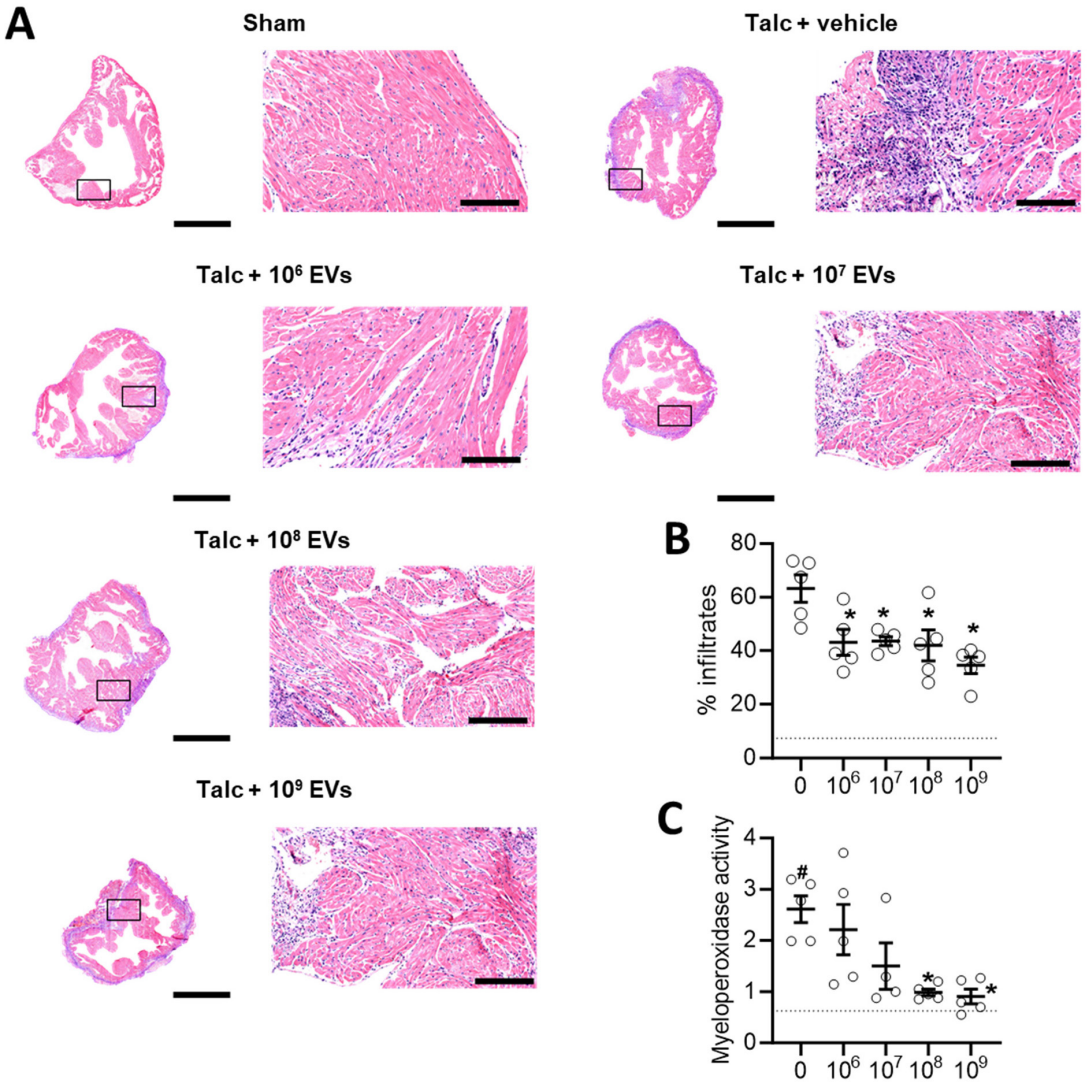
** Intramyocardial injection of human extracellular vesicles (EVs) reduce inflammatory infiltrates and oxidative stress in a model of rat sterile pericarditis.** (A) Representative images from sham, talc + vehicle and talc + EV treated animals showing inflammatory infiltrates using hematoxylin and eosin staining. Scale bar 2 or 0.2 mm, indicated. (B) Random field analysis showing that increasing EV dose had an immediate and progressive inhibitory effect on inflammatory infiltrates (hematoxylin and eosin staining) within talc treated animals (n=5). Dotted line indicates baseline (sham). (C) Effect of pericarditis and EV treatment on myeloperoxidase activity (n=5). One-way ANOVA with individual-mean comparisons by Šídák's multiple comparisons test. *P<0.05 vs. talc + vehicle treated animals. #P<0.05 vs. sham. EV, extracellular vesicles.

Local inflammation is a risk factor for alterations in atrial conduction and postoperative AF 46. Previously, we found that EVs decrease infiltration of immune cells into the atria while also decreasing the abundance of pro-inflammatory cytokines. As shown in Figures 3A-3B and Figure S1, the decrease in immune cell infiltration occurred immediately upon exposure to EVs and progressively decreased with increasing doses. Oxidative stress also plays a key role in the development of postoperative AF with increases in atrial myeloperoxidase activity often associated with increased neutrophil activation, inflammation, and an increased risk of AF 46, 47. Figure 3C demonstrates that administration of talc resulted in an elevation of myeloperoxidase activity in the atrial tissue. However, this increased activity was mitigated by treatment with EVs.

**Figure 4:**
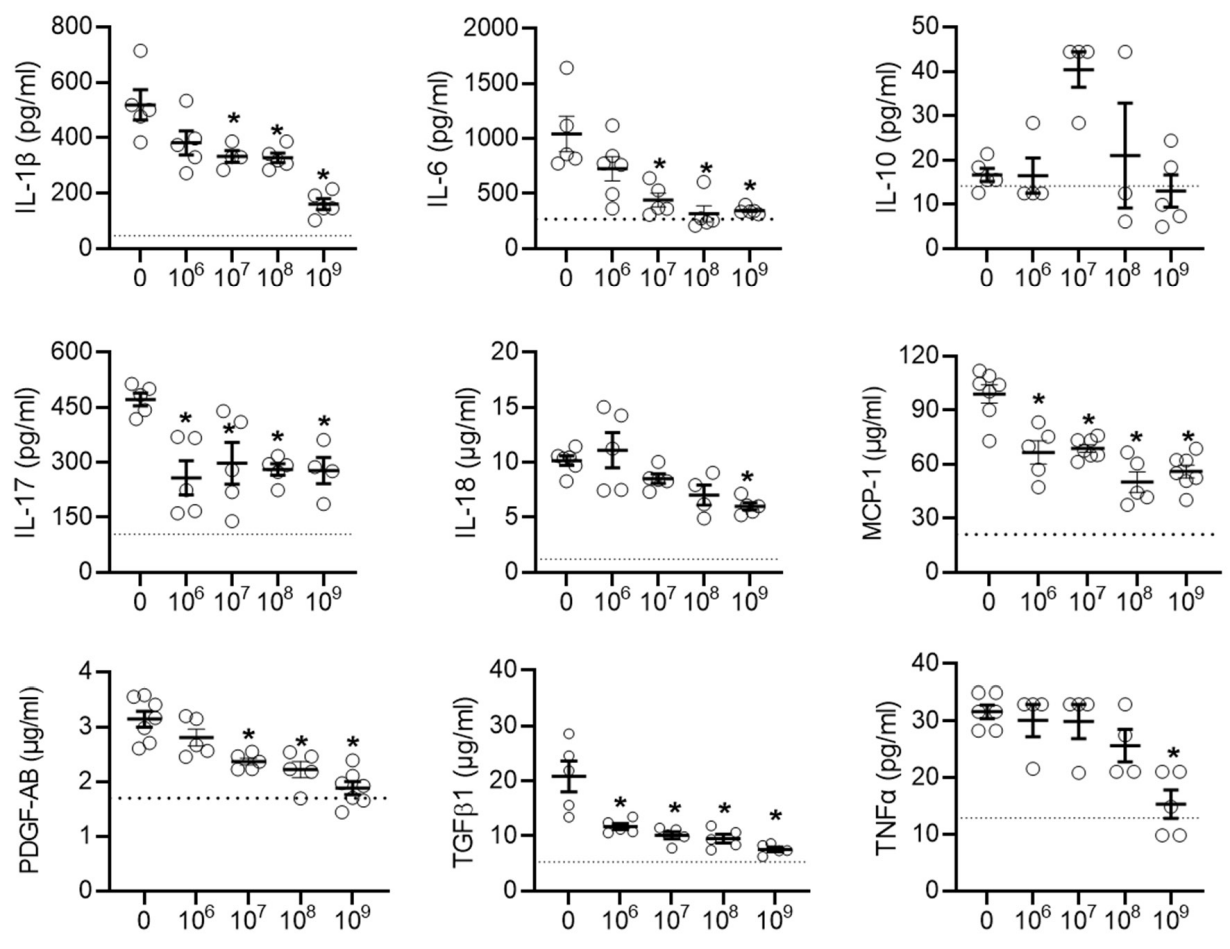
** Intramyocardial injection of human extracellular vesicles (EVs) reduced inflammatory cytokine content in a model of rat sterile pericarditis.** Effect of increasing EV doses on cytokine levels within atrial tissue (n=3-5). One-way ANOVA with individual-mean comparisons by Šídák's multiple comparisons test. *P<0.05 vs. talc + vehicle. IL, interleukin; monocyte chemoattractant protein 1, MCP-1; platelet-derived growth factor AB, PDGF-AB; transforming growth factor beta 1, TGFβ1; tumor necrosis factor alpha, TNFα.

This effect was mirrored by many of the pro-inflammatory cytokines found in the talc treated animals after exposure to EVs (Figure 4). Interestingly, the early cytokine changes observed in atrial tissue after treatment with EVs included several pro-inflammatory cytokines known to play a role in the progression of postoperative AF (IL-6, MCP-1, and TGFβ1) 48, 49. Notably, unlike another type of heart-derived cell 50, EDC EVs had no effect on the cardiac content of IL-10, which has been demonstrated to inhibit the development of AF 51-53.

### Effect of increasing EV doses on atrial remodeling

**Figure 5:**
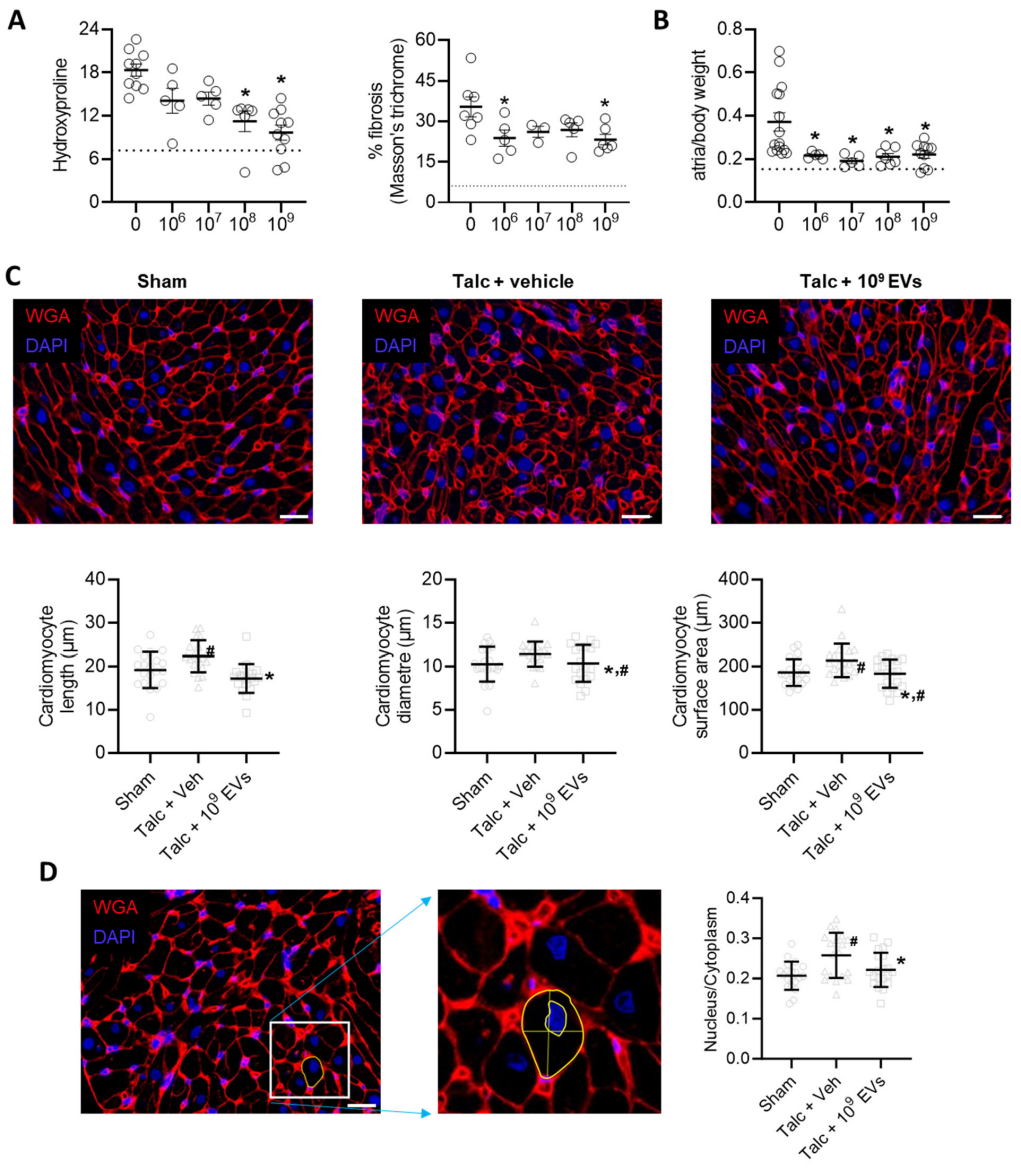
** Intramyocardial injection of human extracellular vesicles (EVs) reduce fibrosis and cardiomyocyte size in a model of rat sterile pericarditis.** (A) Effect of pericarditis and EV treatment on hydroxyproline content (n=5-10) and Masson's Trichrome fibrosis (n=3-7). (B) Effect of pericarditis and EV treatment on atrial mass normalized to body weight (n=5-15). (C) Effect of pericarditis and intramyocardial injection of 10^9^ EVs on cardiomyocyte size quantified using histological analysis of plasma membrane border (n=4 biological replicates with 5 technical replicates) after staining with wheat germ agglutinin (WGA) and 4′,6-diamidino-2-phenylindole (DAPI). Scale bar 20 μm. (D) Effect of pericarditis and intramyocardial injection of 10^9^ EVs on the nucleus to cytoplasm ratio quantified using histological analysis (n= 5 random fields with 10 cells per field) after staining with WGA and DAPI. Scale bar 20 μm. One-way ANOVA with individual-mean comparisons by Šídák's multiple comparisons test. *P<0.05 vs. talc + vehicle. #P<0.05 vs. sham. EV, extracellular vesicles; veh, vehicle.

**Figure 6:**
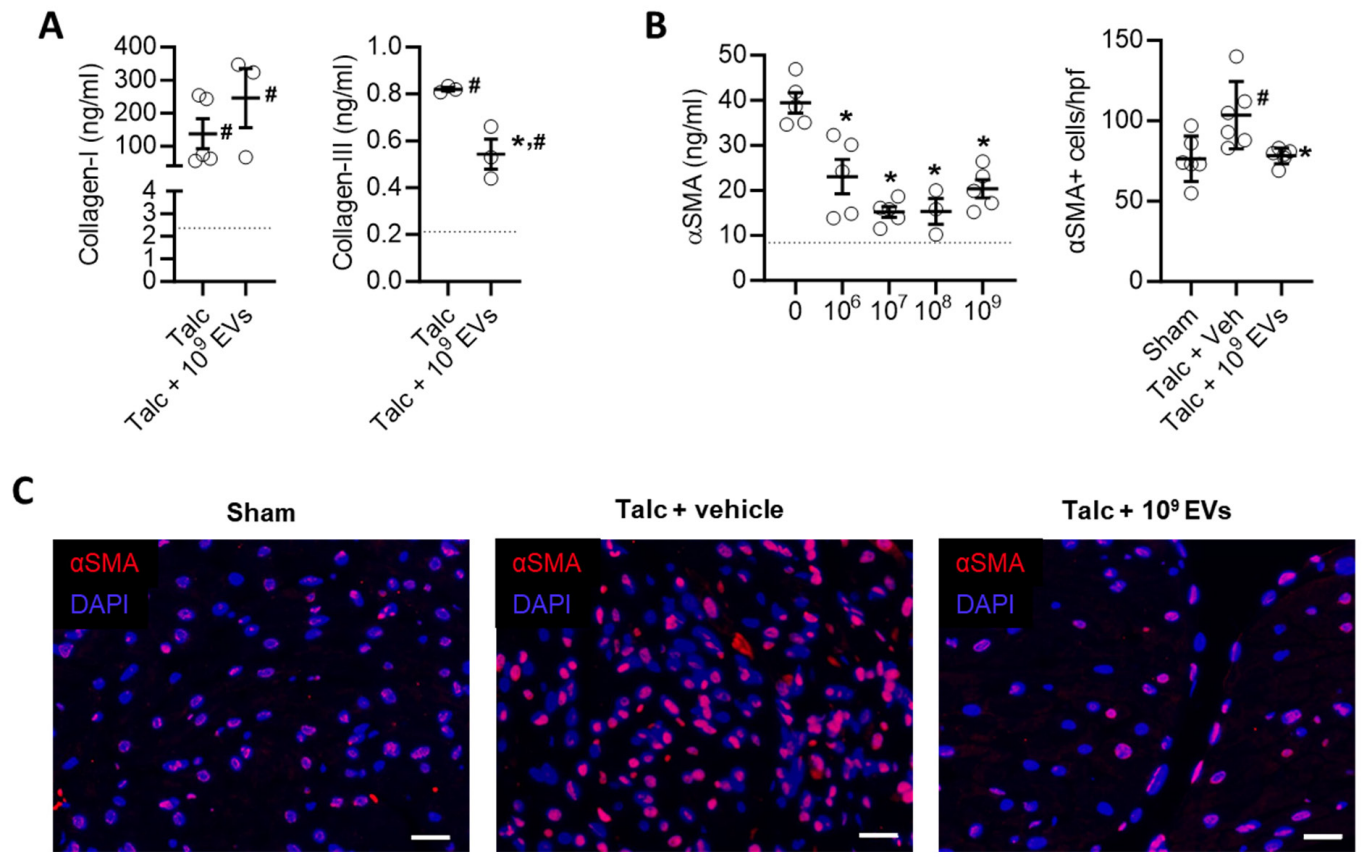
** Intramyocardial injection of human extracellular vesicles (EVs) reduce collagen complexity and pro-fibrotic myofibroblasts within the atria of a model of rat sterile pericarditis.** (A) Effect of pericarditis and EV treatment on collagen I and collagen III content (n=3). (B) Effect of pericarditis and EV treatment on alpha smooth muscle actin (αSMA) content within treated atria (left panel) and the number of αSMA+ cells (n=5). (C) Representative images of the effect of pericarditis and EV treatment on αSMA+ cells. Scale bar 20 μm. One-way ANOVA with individual-mean comparisons by Šídák's multiple comparisons test. *P<0.05 vs. sham animals, #P<0.05 vs. talc + vehicle treated animals. EV, extracellular vesicles; DAPI, 4′,6-diamidino-2-phenylindole; veh, vehicle.

In a manner consistent with other reports 41, 44, 54, pericarditis increased atrial fibrosis by 2.5±0.3 fold (hydroxyproline content, p<0.05 vs sham) and 5.8±1.6 fold (Masson's Trichrome fibrous tissue, p<0.05 vs sham; Figure 5A and Figure S2). Pericarditis also doubled the mass of the left atrial chamber as indicated by the ratio of atrial to body weight (1.0±0.3 fold greater, p<0.05 vs. sham, Figure 5B). This increase was partially attributable to a combination of increased myocyte size (Figures 5C,D) and increased collagen content/complexity (Figure 6A) but may also reflect an increase in inflammatory infiltrates (outlined above).

EV treatment limited the effect of pericarditis on both measures of atrial fibrosis in a dose dependent manner with maximal effects seen after treatment with 10^9^ EVs. Interestingly, the effect of EVs on atrial mass had maximal effects seen after treatment with 10^8^ or 10^9^ EVs (Figures 5A,B). EVs also reduced myocyte size and the ratio of the nucleus to cytoplasm back to baseline (Figures 5C,D). These changes reflected atrial content of profibrotic collagen-secreting myofibroblasts as indicated by the overall αSMA content within atrial tissue and the number of αSMA+ cells (Figures 6B,C).

### EDC EVs attenuate activation of the NLRP3 inflammasome

**Figure 7:**
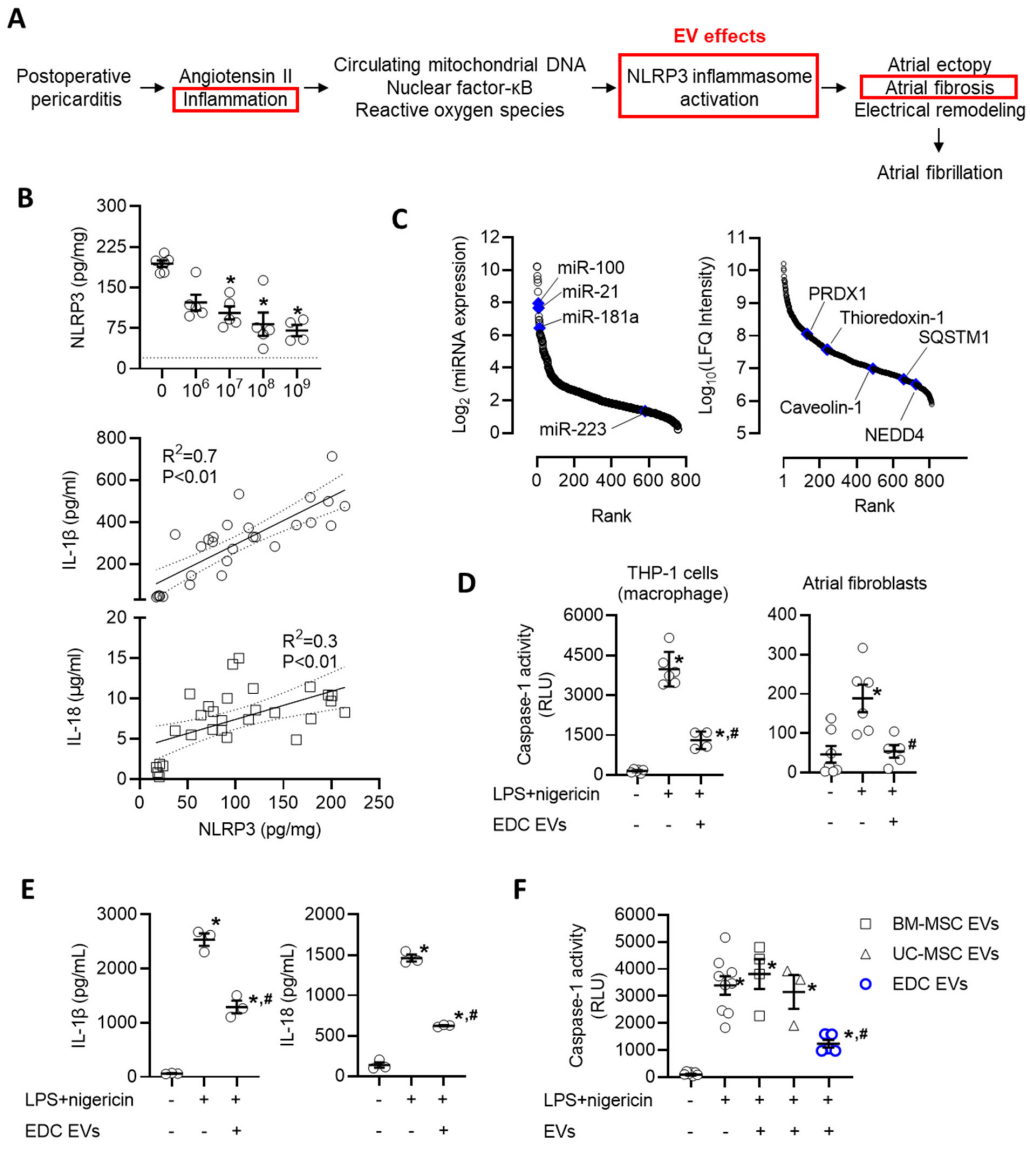
** Intramyocardial injection of human extracellular vesicles (EVs) reduce activation of the nucleotide-binding domain, leucine-rich-containing family, pyrin domain-containing-3 (NLRP3) inflammasome in a model of rat sterile pericarditis.** (A) Schematic outline of the inflammatory effect of post operative pericarditis on NLRP3 activation, atrial remodeling and EV effects (red boxes). (B) Effect of pericarditis and EV treatment on the atrial content of NLRP3 and downstream mediators (interleukin-1 beta (IL-1β) and IL-18). NLRP3 data was analyzed using a one-way ANOVA with individual-mean comparisons by Šídák's multiple comparisons test. *P<0.05 vs. talc + vehicle treated animals. Relationship between NLRP3 and IL-1β or IL-18 abundance within atrial tissue was analyzed using simple linear regression. (C) Rank order plot highlighting the abundance of miRNAs (left panel) and proteins (right panel) found in EVs suggested to directly inhibit NLRP3 activation. (D) In vitro effect of explant-derived cell (EDC) EVs on caspase-1 activity, a measure of NLRP3 activation, in THP-1 cells (macrophages) and primary cultured atrial fibroblasts after activation by lipopolysaccharide (LPS) and nigericin. One-way ANOVA with individual-mean comparisons by Šídák's multiple comparisons test. *P<0.05 vs. untreated cells; #P<0.05 vs. LPS+nigericin treated cells. (E) In vitro effect of EDC EVs on downstream mediators of NLRP3 activation (IL-1β and IL-18) in THP-1 cells (macrophages) after activation by LPS and nigericin. One-way ANOVA with individual-mean comparisons by Šídák's multiple comparisons test. *P<0.05 vs. untreated cells; #P<0.05 vs. LPS+nigericin treated cells. (F) Comparative effect of EVs from bone marrow mesenchymal stromal cells (BM-MSCs), umbilical cord mesenchymal stromal cells (UC-MSCs) or EDCs on caspase-1 activity, the principal effector of NLRP3 activation. One-way ANOVA with individual-mean comparisons by Šídák's multiple comparisons test. *P<0.05 vs. untreated cells; #P<0.05 vs. BM-MSC or UC-MSC treated cells. NEDD4, neural precursor cell expressed developmentally down-regulated protein 4; PRDX1, Peroxiredoxin-1; SQSTM1, Sequestosome 1.

Recent work has implicated activation of the NLRP3 inflammasome system as a key driver in the development of postoperative AF 55-58. Postoperative increases in angiotensin II, circulating mitochondrial DNAs, and local inflammation lead to increases in nuclear factor kappa-light-chain-enhancer of activated B cells (NF-κB) and reactive oxygen species that prime and activate the NLRP3 inflammasome to exacerbate atrial ectopy and promote fibrosis (Figure 7A) 55, 59-61. These changes were reflected within our rat model as pericarditis increased the atrial content of NLRP3 by 8.7±0.7 fold (p<0.05 vs. sham, Figure 7B). Application of EVs resulted in an immediate decrease in NLRP3 which progressively declined with increasing EV doses. Consistent with changes in NLRP3 activation, this effect was paralleled by reductions in IL-1β and IL-18, which represent downstream mediators of NLRP3 activation.

We then questioned if the effect of EVs on NLRP3 represented a direct action on target cells or was merely the indirect result of altering upstream activators/mediators. New bioinformatic analysis on the RNA and protein cargo within EVs suggested the former as the miRNA and protein cargo within EVs included 22 miRNAs and 4 proteins predicted to inhibit NLRP3 (Figure 7C). Inspired by this promising data, we explored the influence of EVs on primary cultured atrial fibroblasts and macrophages (differentiated THP-1 cells) after priming (LPS) and activation (nigericin) of the NLRP3 inflammasome. Using a bioluminescent assay indicative of secreted caspase 1 activity (a direct downstream mediator of NLRP3), we found that EV treated atrial fibroblasts and macrophages displayed significantly attenuated NLRP3 activity as compared to LPS+nigericin-only treated cells (50% lower, p=0.02, Figure 7D). Similar to effects seen in vivo, reduced NLRP3 activation was associated with other downstream mediators of NLRP3 activation (Figure 7E). This suppressive effect was exclusive to EDC EVs as application of EVs from other potentially therapeutic EV sources (i.e., BM-MSCs or UC-MSCs) did not alter NLRP3 activity (Figure 7F).

## Discussion

Decades of work have shown that postoperative AF can be conceptualized as a predictable disease that occurs 2-3 days after cardiac surgery in 20-50% of patients 56. Transient perioperative insults combine with pre-existing and surgery-induced changes to increase atrial vulnerability over a theoretical threshold such that autonomic nervous system activation, inflammation and oxidative stress result in ectopic triggers that enter an adversely remodelled atrial substrate to result in AF. Previously, we used heart explant-derived cells as an ex vivo producer cell line to generate a defined EV product that contains an anti-fibrotic and anti-inflammatory cargo of miRNAs and proteins 24, 29. When 10^9^ EVs were injected directly into the atrial wall at the time of open chest surgery, atrial inflammation and fibrosis was reduced. In this report, we show that EVs provide a dose dependent reduction in inflammation and fibrosis with maximal effects seen after 10^9^ EVs. Mechanistically, we observed that changes in inflammatory cytokine abundance and markers of tissue remodeling lagged behind suppressive effects seen on the NLRP3 inflammasome, a critical driver in the development of postoperative AF 55-58.

These observations highlight the fascinating interplay between structural disease and electrophysiological function that underlies AF. In our review of preclinical biological therapies for AF, we found a mix of approaches that included disrupting cholinergic signaling, altering atrial electrophysiology, reducing apoptosis, and reducing atrial fibrosis 62. Collectively, these interventions reduced the likelihood of inducing AF by 85% (odds ratio 0.15; 95% CI 0.07-0.35; p<0.01). In our 2020 follow-up paper, we focused directly on all pre-clinical therapies for postoperative AF and found approaches that reflected biological (n=5), dietary (n=2), surgical (n=2), and drug (n=17) interventions 63. Successful prevention of postoperative AF uniformly targeted fibrosis or inflammation.

This analysis also revealed that the ideal therapy for postoperative AF needs to be affordable, effective, and non-toxic. When examined in this light, many of the approaches chosen to date fall short. The poor efficacy of antiarrhythmic drugs suggests that changing cellular electrophysiology alone is not likely to be effective. Particularly because this approach treats the pro-arrhythmic remodeled atria alone and does nothing to prevent pathological evolution. Anti-fibrotic and anti-inflammatory approaches may be effective if confined to the atria but are unlikely to have much benefit if they impact postoperative healing or increase the risk of infection. Local delivery of a treatment to the atria avoids many of these systemic issues. Unlike a therapy for longstanding paroxysmal or persistent AF, this injectate must persist long enough to modify cell function but need only be present during the recovery period. A strategy that involves local injection of miRNAs at the time of cardiac surgery to modify cellular electrophysiology or fibrosis for a few days would exemplify this approach. But current delivery systems, such as lipid nano particles (common for RNA delivery), are inherently proinflammatory which rationalizes use as vaccine adjuvants (e.g., COVID-19 vaccinations) but limits application when inflammation plays a role in the pathogenesis.

Increasing EV dose provided a dose dependent reduction in many of the markers implicated in causing postoperative AF. An observation that establishes a biological gradient between EV dose and effect which is useful to infer causality. It follows that EVs need to be administered as a drug to attain therapeutic levels. These effects combined to render the atria resistant to the pro-arrhythmic effects of open chest surgery and did not increase the abundance of factors associated with reducing the incidence of postoperative AF (i.e., IL-10). The latter opens hints at new opportunities to improve the therapeutic payload within EVs as gene editing or somatic gene transfer of producer cell lines may offer new opportunities to enhance the therapeutic payload of the EVs they produce (e.g., over-expression of NT4 to increase IL-10 expression within macrophages that uptake gene modified EVs) 50.

At low doses, EVs appear to act through direct and indirect inhibition of the NLRP3 pathway. Previous work has shown that activation of NLRP3 by angiotensin II leads to macrophage recruitment and the development of fibrosis 60. Similarly, genetic inhibition of NLRP3 has been shown to prevent spontaneous AF in mouse models of AF 57. These findings also have bearing on postoperative AF. Analysis of atrial tissue biopsies taken before surgery has shown abnormalities and activation of NLRP3 inflammasome signaling are evident in patients who subsequently develop postoperative AF. Our rat model of sterile pericarditis extends this paradigm as application of talc increased NLRP3 activation, suggesting that postoperative inflammation exacerbates NLRP3 activation and atrial tissue remodeling. Recently, Yang and colleagues showed that genetic knock out or small molecule blockade of the transient receptor potential vanilloid 4, non-selective cation channel widely expressed in a wide variety of organs, prevented NLRP3 activation and postoperative AF 51. In this study, EVs prevented NLRP3 activation at low doses which is consistent with the observation that EVs contain several proteins and transcripts known to inhibit to NLRP3 activity.

EV effects on fibrosis convincingly occurred only at higher doses (10^8^ and 10^9^) which likely represent a combination of NLRP3 suppression and a direct inhibitory effect on atrial fibroblast proliferation. Previously, we demonstrated that EVs possess many miRNA transcripts known to reduce fibrotic atrial remodeling (miR-26 and miR-29) 64, 65 but, given the complex cargo found within EVs, it is challenging to attribute the observed effect entirely to one transcript or protein. The salutary effects of EVs on fibrosis and inflammation likely combined to reduce AF. Recent reviews of preclinical approaches to AF identified that, except for therapies that manipulated electrophysiological remodelling, all successful approaches targeted either fibrosis or inflammation 62, 63. Future work will be needed to dissect the contribution of fibrosis from inflammation to define the EV anti-arrhythmic mechanism, but it appears clear that EVs address the two fundamental mechanisms repeatedly identified as driving AF.

This study has several important limitations, that include: 1) Reliance upon a rat model of sterile pericarditis. Although murine models are routinely used to explore the mechanism underlying atrial arrhythmias, they cannot fully replicate the complex properties of human AF. As such, validation of EV effects in human models of AF are needed to have confidence in the clinical translational potential of the study findings. 2) Unlike the clinical reality, our rat model of sterile pericarditis does not exhibit pre-established fibrosis or other competing medical comorbidities when subjected to the pro-inflammatory pericarditis insult. This absence is crucial when considering the impact of EVs on the role of pre-existing atrial remodeling in post-operative AF, which is driven by factors such as age, a history of AF, hypertension, left atrial enlargement, and left ventricular dysfunction 56. As such, we may be over-estimating the contribution of inflammation to treatment outcomes. Future work is needed to confirm if the antifibrotic and anti-inflammatory study findings are applicable in models of pro-fibrillatory fibrotic remodelling. 3) Although we have identified several candidate miRNAs and proteins implicated in supressing NLRP3 activation, the specific mediators underlying this effect are not clear. EVs are complex molecules that likely inhibit NLRP3 activation at multiple levels within the pathway. Future work is needed to dissect and confirm if the candidates found within EVs are capable of altering inflammation. 4) Lastly, it is important to acknowledge the limitations inherent in using THP-1 cells as a macrophage model. While THP-1 cells are useful in cardiovascular research and offer insights into macrophage-related mechanisms 66-70, they do not fully capture the complexity of resident or infiltrative cardiac macrophages 71, 72. Thus, the findings derived from this model may not be entirely generalizable to the intricate biology of macrophages in situ, representing an avenue for future work.

## Supplementary Material

Supplementary figures and table.

## Abbreviations

αSMA: alpha smooth muscle actin
AF: atrial fibrillation
BM-MSC: bone marrow mesenchymal stromal cell
CISS: cellular immunotherapy for septic shock
DAPI: 4′,6-diamidino-2-phenylindole
EDC: explant-derived cell
ELISA: enzyme-linked immunosorbent assay
EPS: electrophysiological study
EV: extracellular vesicle
H&E: hematoxylin and eosin
IL: interleukin
LPS: lipopolysaccharide
MCP-1: monocyte chemoattractant protein-1
MMP: matrix metalloproteinase
NEDD4: neural precursor cell expressed developmentally down-regulated protein 4
NF-κB: nuclear factor kappa-light-chain-enhancer of activated B cells
NLRP3: nucleotide-binding domain, leucine-rich-containing family, pyrin domain-containing-3
PDGF-AB: platelet-derived growth factor AB
PRDX1: peroxiredoxin-1
SQSTM1: sequestosome 1
TGFβ1: transforming growth factor beta 1
TIMP: tissue inhibitor of metalloproteinase
TNFα: tumor necrosis factor alpha
UC-MSC: umbilical cord mesenchymal stromal cell
veh: vehicle
WGA: wheat germ agglutinin
