# Supplementary material for: Inactivation of the NLRP3 inflammasome mediates exosome-based prevention of atrial fibrillation
Source: Theranostics. 2024 Jan 1;14(2):608–21. doi: 10.7150/thno.89520 (PMC10758048; doi:10.7150/thno.89520)
Supplement: Supplementary file 1 — Supplementary figures and table. [file thnov14p0608s1.pdf]

	P wave (ms)	Heart rate (bpm)	PR (ms)	QRS (ms)	QTc (ms)	AERP (ms)	AVERP (ms)	AF incidence (%)
Sham (n=14)	16±1	349±18	49±3	19±2	224±8	37±5	77±5	16%
Sham + EVs (n=4)	15±2	348±22	45±3	18±1	211±7	43±3	80±4	25%
P value	0.71	0.97	0.2	0.62	0.17	0.3	0.53	0.42

Supplemental Table 1 – Electrocardiographic and electrophysiological effects of intramyocardial injection of extracellular vesicle (EV) into normal hearts compared to sham animals. AERP, atrial effective refractory period; AF, atrial fibrillation; AVERP, atrioventricular effective refractory period. Data presented as mean ± standard error of the mean. Continuous variables were analyzed using students T-test while categorical variables were analyzed using Fisher's exact test.

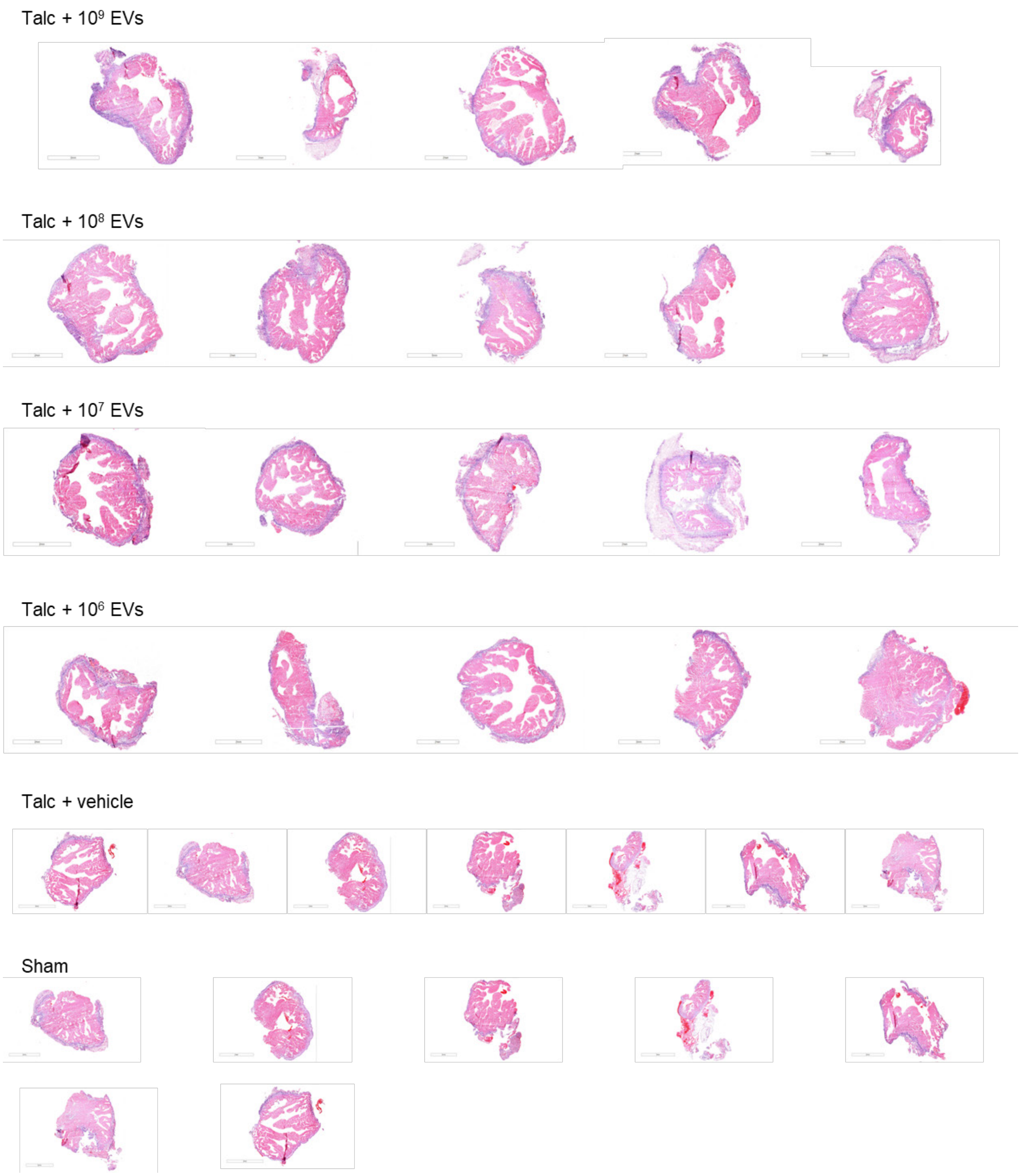

Supplemental Figure 1 – Effect of extracellular vesicles (EVs) on atrial inflammatory infiltrates. Hematoxylin and eosin images from each animal treated are shown demonstrating the effect of pericarditis and EVs on atrial inflammatory infiltrates. EV, extracellular vesicle. Scale bars 2mm.

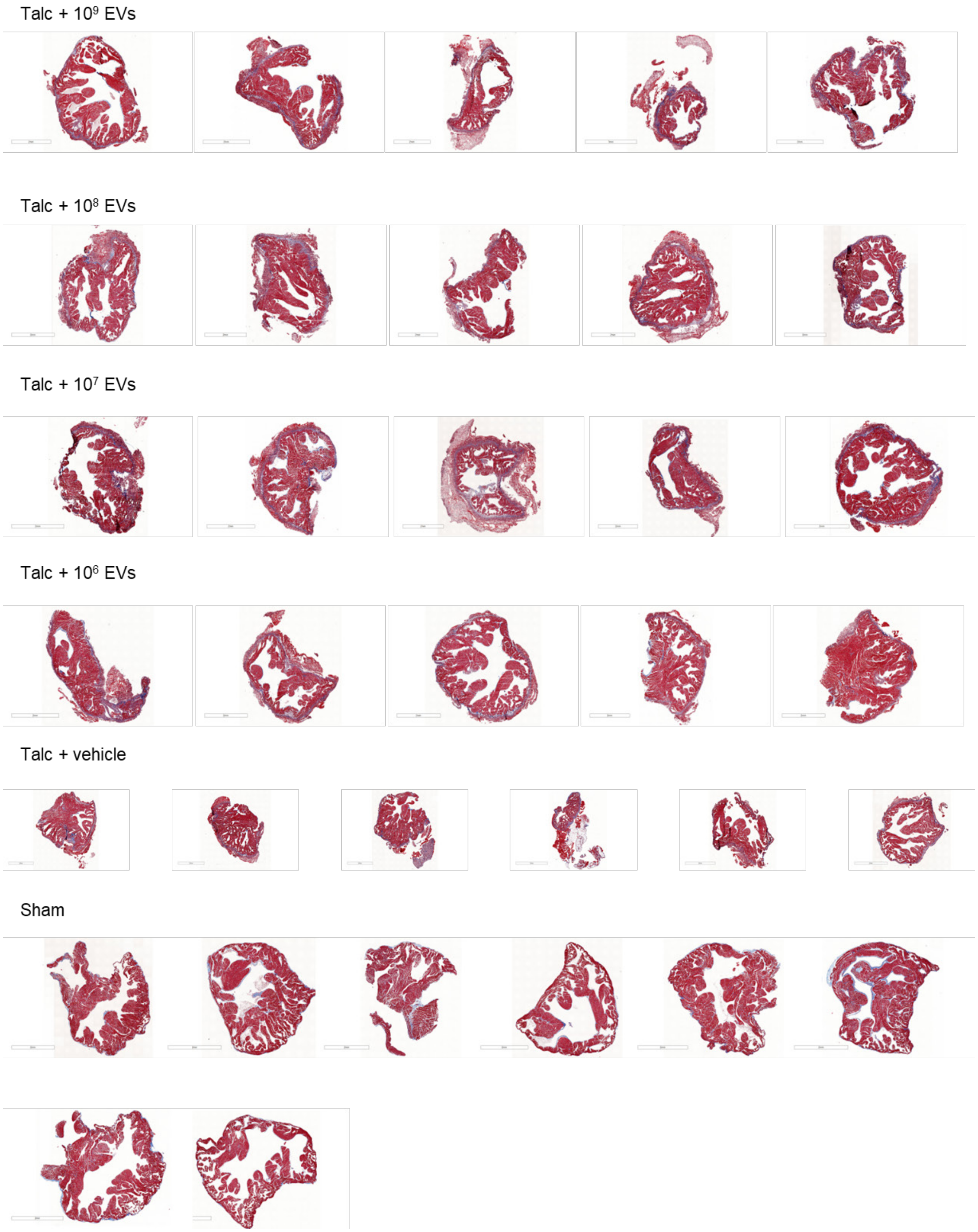

Supplemental Figure 2 – Effect of extracellular vesicles (EVs) on atrial fibrosis. Masson's Trichrome images from each animal treated are shown demonstrating the effect of pericarditis and EVs on atrial fibrosis. EV, extracellular vesicle. Scale bars 2mm.
